# Inflammation Triggers Emergency Granulopoiesis through a Density-Dependent Feedback Mechanism

**DOI:** 10.1371/journal.pone.0019957

**Published:** 2011-05-31

**Authors:** Derek W. Cain, Pilar B. Snowden, Gregory D. Sempowski, Garnett Kelsoe

**Affiliations:** 1 Department of Immunology, Duke University, Durham, North Carolina, United States of America; 2 Duke University Human Vaccine Institute, Duke University Medical Center, Durham, North Carolina, United States of America; National Institute of Environmental Health Sciences, United States of America

## Abstract

Normally, neutrophil pools are maintained by homeostatic mechanisms that require the transcription factor C/EBPα. Inflammation, however, induces neutrophilia through a distinct pathway of “emergency” granulopoiesis that is dependent on C/EBPβ. Here, we show in mice that alum triggers emergency granulopoiesis through the IL-1RI-dependent induction of G-CSF. G-CSF/G-CSF-R neutralization impairs proliferative responses of hematopoietic stem and progenitor cells (HSPC) to alum, but also abrogates the acute mobilization of BM neutrophils, raising the possibility that HSPC responses to inflammation are an indirect result of the exhaustion of BM neutrophil stores. The induction of neutropenia, via depletion with Gr-1 mAb or myeloid-specific ablation of Mcl-1, elicits G-CSF via an IL-1RI-independent pathway, stimulating granulopoietic responses indistinguishable from those induced by adjuvant. Notably, C/EBPβ, thought to be necessary for enhanced generative capacity of BM, is dispensable for increased proliferation of HSPC to alum or neutropenia, but plays a role in terminal neutrophil differentiation during granulopoietic recovery. We conclude that alum elicits a transient increase in G-CSF production via IL-1RI for the mobilization of BM neutrophils, but density-dependent feedback sustains G-CSF for accelerated granulopoiesis.

## Introduction

C/EBPα and -β play critical roles in regulating the tempo of granulopoiesis. Neutrophil pools in the blood and BM are normally maintained by a granulopoietic process dependent on the C/EBPα transcription factor [1], [2]. In contrast, inflammation accelerates granulopoiesis and induces neutrophilia by a distinct form of granulopoiesis acting through a C/EBPβ-dependent pathway [2], [3]. C/EBPβ is thought to facilitate proliferation by myeloid progenitor cells in response to inflammatory growth factors [2], but the extracellular signals that stimulate neutrophil production during inflammatory responses are unclear.

Mice with a conditional deletion of C/EBPα fail to produce neutrophils due to defective generation of granulocyte/macrophage progenitors (GMP) from common myeloid progenitors (CMP) [4]. Granulopoiesis can be rescued in C/EBPα^−/−^ mice by exogenous GM-CSF or IL-3 [2], or by the expression of C/EBPβ from the C/EBPα locus [5]. C/EBPβ^−/−^ mice, in contrast, exhibit normal numbers of neutrophils in the steady-state, but fail to mount neutrophilias in response to infection or cytokine treatment, implicating C/EBPβ as a regulator of emergency granulopoiesis [2]. Growth factors elicited during infections are thought to reduce C/EBPα expression in GMP while increasing C/EBPβ expression, and thereby focusing differentiation towards neutrophils while elevating rates of proliferation [2]. The model that has emerged from these observations is that C/EBPα and C/EBPβ control distinct pathways of steady-state and emergency granulopoiesis, respectively.

While neutrophilias are commonly associated with inflammation, it is generally thought that granulopoiesis is a dynamic process that also responds to non-inflammatory cues elicited through feedback. Evidence for feedback mechanisms of granulopoiesis was first provided in studies showing that animals rendered neutropenic via leukapheresis [6], neutrophil anti-serum [7], or irradiation [8] exhibited expansions of myeloid precursor populations in BM prior to the recovery of mature neutrophil compartments. In patients with cyclic neutropenia, the periodic oscillations in blood neutrophil counts are thought to result from dysfunctional feedback regulation of granulopoiesis [9]. Numerous models for granulopoietic regulation have been proposed and most contain a feedback component that links the population density of neutrophils to the proliferation and differentiation of neutrophil precursors [10], [11], [12], [13]. The mechanisms of feedback are still debated, however, and the roles of C/EBPα and C/EBPβ in the active regulation of granulopoiesis have not been addressed.

Several cytokines can modulate neutrophil production, but G-CSF is the primary regulator of steady-state and emergency granulopoiesis (reviewed in [14]). In addition to regulating neutrophil output, signaling through G-CSF and G-CSF receptor (G-CSF-R) also controls neutrophil survival, function, and egress from BM [15], [16], [17]. Consequently, mice lacking G-CSF or G-CSF-R are severely neutropenic [15], [16]. G-CSF-independent pathways of granulopoiesis exist, however, as mature neutrophils are present in both G-CSF^−/−^ and G-CSF-R^−/−^ mice, albeit in lower numbers [15], [16]. Furthermore, G-CSF is dispensable for the inflammatory neutrophilia elicited by fungal infection [18], indicative of a compensatory signal(s) that enhances granulopoiesis in response to pathogens. Candidate factors for the inflammatory induction of granulopoiesis include GM-CSF, IL-3, and IL-6 [19], [20], [21], [22]. Microbial products may also enhance myelopoiesis directly, as TLR ligation on HSPC induces proliferation and myeloid differentiation *in vitro* [23].

Recently, we demonstrated that the sterile adjuvant alum induces emergency granulopoiesis by increasing the proliferation of hematopoietic stem cells (HSC), multipotent progenitors (MPP), and granulocyte/macrophage progenitors (GMP). In IL-1RI^−/−^ mice, alum does not increase the proliferation of HSPC [3]. However, the IL-1RI sensitive compartment in these mice is not HSPC but a radiation-resistant cell population that is not transferred by hematopoietic reconstitution [3]. Therefore, IL-1RI signaling must elicit another factor that activates HSPC for accelerated neutrophil production.

Here, we show that alum rapidly elicits G-CSF via IL-1RI-dependent signals. Interference with G-CSF/G-CSF-R abrogated the inflammatory mobilization of BM neutrophils and increases in HSPC proliferation, raising the possibility that increased HSPC proliferation is an indirect effect of reduced BM neutrophil numbers. Indeed, *in vivo* depletions of neutrophils stimulated HSPC proliferation and emergency granulopoiesis as well as alum. We conclude that inflammation rapidly induces G-CSF for the mobilization of BM neutrophils, but the resulting BM neutropenia triggers increases in HSPC proliferation through a density-dependent feedback mechanism. Notably, the transcription factor C/EBPβ, thought to be necessary for enhancing the generative capacity of bone marrow, is dispensable for increased proliferation of HSPC but plays a role in terminal neutrophil differentiation during granulopoietic recovery from neutropenia. These observations reveal the BM neutrophil population density as a key regulator of neutrophil production; inflammation mobilizes BM neutrophils, effecting neutropenia which, in turn, activates a density-dependent feedback mechanism for increased HSPC proliferation and focused neutrophil production.

## Materials and Methods

### Ethics Statement

All studies were approved by the Duke University Institutional Animal Care and Use Committee (permit number 240-08-09), and every effort was taken to minimize animal suffering.

### Mice

C57BL/6, congenic IL-1RI^−/−^ mice (B6.129S7-Il1r1^tm1Imx^/J [24]), and RAG1^−/−^ mice (B6.129S7-Rag1^tm1Mom^/J[25]) were from Jackson Laboratories. Dr. Y. Yang (Duke University) provided MyD88^−/−^ mice [26]. C1q^−/−^ mice [27] were obtained from Dr. M. Walport (Imperial College). C3^−/−^ mice [28] and FcR common γ chain deficient mice (FcRγ^−/−^ [29]) were provided by Dr. T. F. Tedder (Duke University). LysM^Cre/wt^ Mcl-1^f/f^ and LysM^wt/wt^ Mcl-1^f/f^ mice [30] were provided by Dr. Y. He (Duke University). C/EBPβ hemizygous mice on 129/Sv and BL/6 background strains were provided by Dr. Peter Johnson (NCI); C/EBPβ^−/−^ mice and control littermates were generated by intercrossing hemizygous 129/Sv and BL/6 parents. Bone marrow cells from CX_3_CR1^GFP/+^ mice [31] were provided by Dr. M.D. Gunn (Duke University). G-CSF-R^−/−^ mice [16] were provided by Dr. D. Link (Washington University). All mice were housed in specific pathogen-free conditions at the Duke University Animal Care Facility with sterile bedding, water, and food; MyD88^−/−^ mice were provided with antibiotic in water.

### Purification of Gr-1 mAb

Gr-1 mAb [32] was purified from serum-free supernatants using a HiTrap Protein G column (GE Healthcare) and dialyzed in sterile PBS.

### Injection of adjuvant and purified proteins

Mice were immunized *i.p*. with chicken γ-globulin (CGG) conjugated to (4-hydroxy 3-nitrophenyl)acetyl (NP) (NP_9-11_-CGG) precipitated in alum [33]. Purified Gr-1 mAb or anti-Ly-6G mAb (100 µg, clone 1A8, Biolegend) was diluted to 200 µl in PBS and injected *i.p.* To neutralize G-CSF *in vivo*, mice were injected *i.v.* with 100 µg anti-mouse GCSF (R&D Systems, clone 67604) 30 minutes before immunization or Gr-1 injection.

### Flow Cytometry

FITC-, PE-, PE-Cy5-, APC-, APC-Cy7 or APC-eFluor 780, PE-Texas Red-, PE-Cy7-, and biotin-conjugated antibodies to Ly-6G (clone 1A8), Gr-1, CD11b, Ter119, CD4, CD8, B220, TCRβ, Ly-6C, CD31, CD34, c-Kit, Sca-1, FcγRII/III, and Flt3 were from BD Pharmingen or eBioscience. PE-conjugated Ly-6B mAb was obtained from AbD Serotec and FITC-conjugated anti-CCR3 was obtained from R&D Systems.

Mice were bled via the retro-orbital sinus and blood was collected in heparin solution. Femurs and tibiae from both legs were harvested. BM was flushed out with cold IMDM containing 2% FBS. Erythrocytes were lysed in ammonium chloride buffer. Single-cell suspensions were labeled with combinations of fluorochrome-antibody conjugates; propidium iodide (Sigma-Aldrich) identified dead cells.

As injected Gr-1 mAb reduced levels of *ex vivo* labeling with fluorochrome-Gr-1 conjugates, Gr-1^+^ cells were routinely identified by the following procedure. In a first round of labeling, cells were exposed to unconjugated Gr-1, washed, and saturated with FITC-conjugated anti-rat IgG (Southern Biotech). This method ensured the identification of all Gr-1^+^ cells, whether labeled *in vivo* or *ex vivo*. After washing, labeling with other antibody conjugates was carried out in a second round of staining.

Antibody-labeled cells were analyzed on a LSRII flow cytometer (BD Biosciences) or sorted with a FACSVantage (BD Biosciences). Cell sorting was performed in the Duke Human Vaccine Institute Research Flow Cytometry Shared Resource Facility. Flow cytometric data were analyzed with FlowJo software (Treestar). Cytospins of sorted cells were stained using Hema3 (Fisher Scientific) to confirm their identities (Fig. S1). For analysis of BrdU uptake by HSPC, HSC were identified as Flt3^−^Lin(Gr-1, CD11b, Ter119, CD4, CD8, B220) ^−^c-Kit^+^Sca-1^+^, MPP were identified as Flt3^+^Lin^−^c-Kit^+^Sca-1^+^, and GMP were defined as Lin^−^c-Kit^+^Sca-1^−^FcγRII/III^+^ [3].

### BrdU labeling

HSPC proliferation was determined using a BrdU labeling kit (BD Biosciences). Six hours before sacrifice, mice were injected *i.p.* with 1 mg BrdU in 200 µl PBS. After labeling with fluorochrome-antibody conjugates specific for surface antigens, BM cells were fixed/permeabilized, treated with DNase, and incubated with FITC anti-BrdU.

### Quantification of serum cytokines

Serum cytokines were analyzed in the Duke Human Vaccine Institute Immune Reconstitution & Biomarker Shared Resource Facility. Sera were prepared from naïve BL/6, Mcl-1^+^, Mcl-1^−^ mice, and BL/6 mice injected with alum or 100 µg Gr-1 mAb. Cytokine concentrations in undiluted serum samples were determined using a Bio-Plex Pro Group I 23-Plex kit (Bio-Rad). Readings below the lowest point in the standard curve were mathematically extrapolated, but these measurements were imprecise.

Serum G-CSF was quantified by ELISA. Wells of a 96-well plate were coated overnight with anti-G-CSF (R&D Systems, clone 67604) then blocked for 1 hour with PBS/1%BSA. Serum was added to wells and incubated for 2 hours at room temperature. Bound G-CSF was detected with biotinylated anti-GCSF (R&D Systems) and SA-horseradish peroxidase (Southern Biotechnology). Horseradish peroxidase activity was revealed with a tetramethylbenzadine peroxidase substrate kit (Bio-Rad). G-CSF concentrations in samples were calculated from a standard curve of recombinant mouse G-CSF (Peprotech); the sensitivity of the assay was 10 pg/ml G-CSF.

### Statistics

Significance in paired data was determined by Student's *t*-test. Relationships between cell numbers and the frequencies of BrdU^+^ HSC, MPP, and GMP were evaluated using the Pearson product moment correlation coefficient. Hematopoietic responses of C/EBPβ-sufficient and -deficient mice to Gr-1 or alum treatment were compared using a two-way ANOVA with Tukey post-hoc test.

## Results

### Alum induces HSPC proliferation via IL-1RI-dependent G-CSF induction

**Figure 1:**
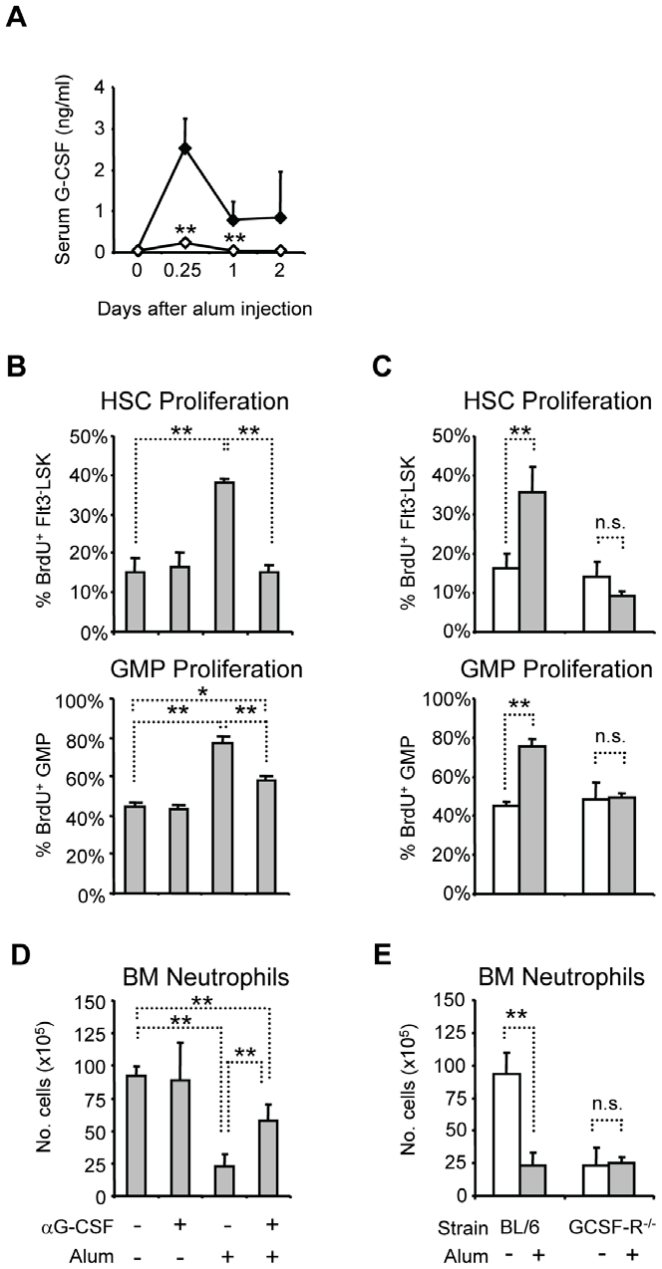
IL-1RI-dependent induction of G-CSF is critical for BM neutrophil mobilization and increased HSPC proliferation after alum immunization. (A) Serum G-CSF concentrations in BL/6 (closed) and IL-1RI^−/−^ (open) mice after alum immunization. The mean(+SD) of serum G-CSF concentration is shown; statistical differences between G-CSF concentrations in BL/6 and IL-1RI^−/−^ mice at each interval are indicated (n = 3–7). (B) Effects of G-CSF neutralization on alum-induced HSC and GMP proliferation. BL/6 mice were treated or not with anti-G-CSF mAb, then immunized or not with alum; mice were sacrificed 24 hours later for analysis. Six hours prior to sacrifice, mice were injected i.p. with BrdU; the mean frequencies(+SD) of BrdU^+^ cells in the HSC and GMP compartments are shown (n = 4–5). (C) Alum immunization of G-CSF-R^−/−^ mice. BL/6 and G-CSF-R^−/−^ mice were immunized with alum (grey) or left untreated (open), and then analyzed for BrdU uptake in HSC and GMP compartments 24 hours later (n = 3–9). (D) Effects of G-CSF neutralization on BM neutrophil mobilization after alum immunization. BM neutrophils were enumerated 24 hours after immunization (Fig. S1 defines neutrophil populations). The mean numbers(+SD) of mature neutrophils in the leg bones are shown for each treatment (n = 4–5). (E) BL/6 or G-CSF-R^−/−^ mice were immunized with alum (grey) or left untreated (open), and the number of BM neutrophils was determined 24 hours later (n = 3–9). *, P≤0.05; **, P≤0.01; n.s., not significant.

Alum elicits reactive neutrophilia by increasing HSC and GMP proliferation through an IL-1RI-dependent pathway [3]. Since G-CSF is an important regulator of granulopoiesis [14], we determined if alum induces G-CSF via IL-1RI by measuring G-CSF concentrations in the serum of immunized BL/6 and congenic IL-1RI^−/−^ mice. In BL/6 mice, G-CSF concentrations rose from 70 pg/ml to 2.5 ng/ml at 6 h after immunization, and remained elevated at 0.7 ng/ml for two days after injection (Fig. 1A). Although serum G-CSF concentrations were equivalent in naïve BL/6 and IL-1RI^−/−^ mice, G-CSF production in response to alum in IL-1RI^−/−^ mice was significantly impaired (Fig. 1A), indicating that alum induces G-CSF via IL-1RI-dependent signals.

To investigate the role of G-CSF in alum-induced HSC and GMP proliferation, we administered a G-CSF neutralizing mAb [34] to BL/6 mice 30 minutes prior to immunization; HSC and GMP proliferation was measured by BrdU incorporation one day after immunization [3]. Anti-G-CSF treatment had no effect on basal HSC and GMP proliferation, but completely abrogated HSC responses to alum (Fig. 1B). In contrast, anti-G-CSF treatment only partially blocked the effects of alum on GMP proliferation (Fig. 1B), suggesting that G-CSF-independent signals generated during the inflammatory response may specifically target GMP to increase proliferation. Alternatively, if G-CSF neutralization is incomplete, GMP may be more sensitive than HSC to residual G-CSF.

We next examined the effects of alum on HSPC proliferation in mice lacking G-CSF-R [16]. In naive G-CSF-R^−/−^ mice, the frequencies of BrdU^+^ HSC and GMP were identical to congenic BL/6 controls (Fig. 1C). The number of HSC in BM of G-CSF-R^−/−^ mice was the same as in BL/6 mice (0.6±0.2×10^5^ HSC in G-CSF-R^−/−^ mice *vs*. 0.6±0.1×10^5^ cells in BL/6 mice, P>0.05), but GMP numbers were reduced by 60% (2.1±0.5×10^5^ GMP in G-CSF-R^−/−^ mice *vs*. 5.0±0.1×10^5^ cells in BL/6 mice, P≤0.01), consistent with previous reports [15], [16]. Although G-CSF-R was dispensable for basal proliferation by HSC and GMP, alum did not increase BrdU uptake by HSC or GMP in G-CSF-R^−/−^ mice (Fig. 1C), indicating that G-CSF-R is necessary for the proliferative responses of HSC and GMP to adjuvant.

G-CSF has well-documented growth factor properties, but it is also a potent mobilizer of BM neutrophils [15], [35]. Indeed, anti-G-CSF treatment significantly impaired neutrophil mobilization by alum (Fig. 1D) and in G-CSF-R^−/−^ mice, alum had no mobilizing effect on residual BM neutrophils (Fig. 1E) (Fig. S1 defines leukocyte populations). Thus, G-CSF/G-CSF-R blockade impairs two components of the inflammatory response that directly contribute to reactive neutrophilia: mobilization of BM neutrophils and increased HSPC proliferation.

### Bone marrow neutropenia by antibody depletion

It is now believed that infections accelerate granulopoiesis through a transcriptional pathway that is distinct from the processes that maintain neutrophil pools in the steady-state, but depends on the inflammatory induction of growth factors [2]. However, emergency granulopoiesis might be an indirect effect of BM neutropenia, as defects in alum-induced HSPC proliferation coincided with failed neutrophil mobilization (Fig. 1 and Ref. 3). Indeed, it has been postulated that the consumption of neutrophil stores during infection might activate feedback mechanisms of neutrophil replenishment [36]. To test the hypothesis that reductions in BM neutrophil numbers trigger emergency granulopoiesis, we studied two experimental models for induced neutropenia that minimize or eliminate ancillary inflammation.

**Figure 2:**
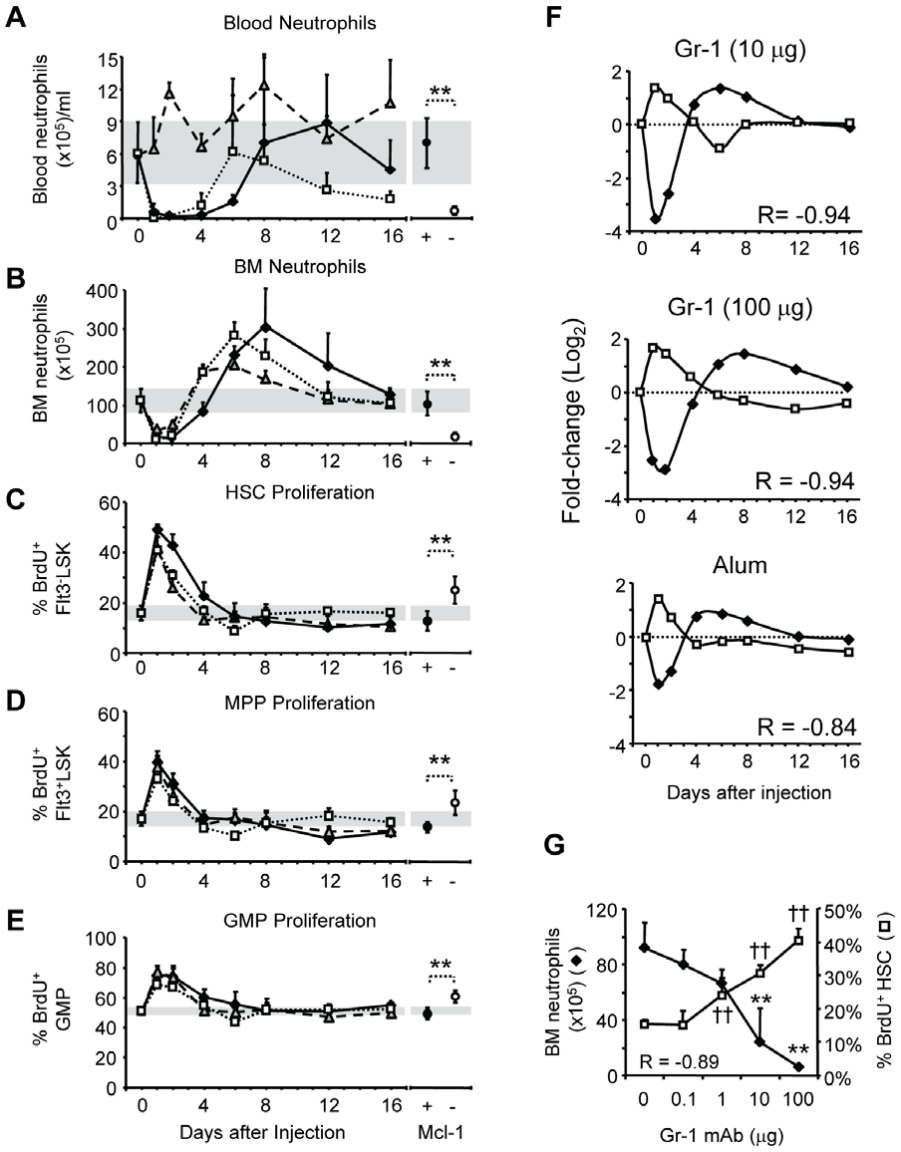
Adjuvant inflammation and experimental neutropenia elicit similar changes in granulopoiesis. BL/6 mice were i.p. injected with Gr-1 mAb [10 µg (open squares), 100 µg (closed diamonds)] or alum (shaded triangles), and then blood and BM neutrophils were enumerated at various intervals. Neutrophils numbers were also determined in Mcl-1^-^ mice and control littermates. The mean(+SD) numbers of neutrophils/ml of blood (A) and in the four leg bones (B) after each treatment are shown. In the right panel, the numbers of blood and BM neutrophils in Mcl-1^+^ and Mcl-1^−^ mice are indicated. Proliferation of HSC (C), MPP (D), and GMP (E) at intervals after alum or Gr-1 treatment, and in Mcl-1^+^ and Mcl-1^−^ mice, was determined by BrdU incorporation. Mice were injected with BrdU 6 hours prior to sacrifice, and the mean frequency(+SD) of BrdU^+^ cells in each compartment is shown (day 0, n = 19; for other intervals, n = 3–10). (F) BM neutrophil numbers (closed diamonds) and frequencies of BrdU^+^ HSC (open squares) after injection of Gr-1 or alum (as shown in B and C) are co-plotted; the values at each interval represent the fold change from naïve mice, expressed as log_2_. Pearson product moment correlation coefficients (R) between BM neutrophil numbers and the frequencies of BrdU^+^ HSC for each treatment are shown. (G) BL/6 mice were injected with graded amounts of Gr-1 mAb (0, 0.1, 1, 10, and 100 µg; n = 4–7 mice per treatment) and then BM neutrophil numbers and HSC proliferation were determined two days later. Significant differences from naïve BL/6 mice are shown for neutrophil numbers (**, P≤0.01) and frequency of BrdU^+^ HSC (^††^, P≤0.01); the Pearson product moment correlation coefficient (R) for BM neutrophil numbers and frequencies of BrdU^+^ HSC is shown.

The Gr-1 mAb is used routinely *in vivo* to deplete neutrophils [37], [38]. We injected BL/6 mice *i.p.* with 10- or 100 µg of Gr-1 mAb and compared the loss and recovery of mature neutrophils in blood and BM to that induced by alum [3]. Whereas alum elicited a multiphasic neutrophilia in blood, both doses of Gr-1 induced transient neutropenias (>90% reductions; P≤0.01) for 2 to 4 days (10- or 100 µg Gr-1, respectively) with recovery on days 6 or 8 (10- or 100 µg Gr-1, respectively) (Fig. 2A). The effects of Gr-1 mAb were specific, as injection of 100 µg rat IgG had no effect on blood or BM leukocyte numbers (unpublished data).

Although alum stimulated neutrophilia and Gr-1 effected neutropenia (Fig. 2A), the two treatments induced similar changes in BM neutrophil populations. Injection of either alum or Gr-1 mAb significantly reduced BM neutrophil numbers for two days (P≤0.01, Fig. 2B); afterwards, BM neutrophil numbers recovered and eventually surged above normal levels on days 6–8 (P≤0.01, all) before gradually returning to normal (Fig. 2B). Notably, the magnitude of the BM neutrophil “rebound” following Gr-1 treatment outpaced that of alum (Fig. 2B), demonstrating that the myeloablative effects of passive Gr-1 mAb are sufficient to induce emergency granulopoiesis.

Given that alum increases proliferation by HSC, MPP, and GMP [3], we determined whether Gr-1 treatment also increased HSPC proliferation. The frequencies of BrdU^+^ HSC, MPP, and GMP increased significantly within one day of Gr-1 administration (P≤0.01, all) and then gradually returned to naïve levels by days 4–6 (Fig. 2C-E). The transient increases in the frequency of BrdU^+^ HSC, MPP, and GMP were mirrored in the cell numbers within each progenitor compartment; cell numbers were elevated when frequencies of BrdU^+^ cells were high, and then gradually to returned to naïve levels (Fig. S2). This observation is consistent with a recent report describing an expansion of hematopoietic progenitor compartments following Gr-1 administration [39]. Notably, administration of 10 µg Gr-1 induced similar proliferative responses in HSPC compartments as alum and BM neutrophil numbers peaked simultaneously (day 6; Fig. 2B). Injection of 100 µg Gr-1 resulted in a longer period of elevated HSC proliferation (Fig. 2C) that was associated with an even greater rebound in BM neutrophil numbers on day 8 (Fig. 2B). Thus, Gr-1 depletion elicits a granulopoietic response that closely mimics that induced by alum, consistent with the possibility that a reduction in BM neutrophil numbers is itself, sufficient to elicit increased HSPC proliferation and accelerated neutrophil production.

Notably, alum elicited a BM eosinophilia in addition to neutrophilia whereas Gr-1 had little effect on BM eosinophil numbers (Fig. S3); inflammation, therefore, more broadly affects hematopoiesis.

Although Gr-1 mAb depleted neutrophils, other hematopoietic lineages were also affected, including Gr-1^-^ B-lineage and erythroid cells (Fig. S3). While we cannot exclude non-specific antibody effects, we suspect that reductions in these cell compartments are secondary to the loss of Gr-1^+^ cells.

### Bone marrow neutropenia by genetic deletion

We also examined a genetic model of neutropenia for evidence of increased HSPC proliferation. Mice with the myeloid-specific deletion of the anti-apoptotic factor Mcl-1 (LysM^Cre/wt^ Mcl-1^f/f^ mice; hereafter Mcl-1^−^ mice) exhibit a highly specific defect in neutrophil survival [30]. Whereas neutrophil numbers in the blood and BM of Mcl-1^−^ mice were only 10% and 20%, respectively, of control littermates (P≤0.01; Fig. 2A, B), the number of developing neutrophils (Fig. S1) was 2-fold greater in Mcl-1^−^ mice (P≤0.05; Table S1). Eosinophil, monocyte, erythroid and B-lineage cell numbers were normal or slightly elevated (Table S1).

Mature neutrophils in Mcl-1^−/−^ mice are lost by a presumably non-inflammatory, apoptotic mechanism [30], [40]. However, HSC, MPP, and GMP proliferation was constitutively increased in neutropenic Mcl-1^−^ mice compared with littermate controls (P≤0.01, all) (Figs. 2C-E) and the numbers of cells in each HSPC compartment were significantly elevated (P≤0.01, HSC and GMP; P≤0.05, MPP)(Fig. S2), consistent with the hypothesis that neutropenia is sufficient to stimulate HSPC proliferation and accelerate neutrophil production.

### Induced proliferation of HSPC is inversely proportional to BM neutrophil numbers

The reciprocal association between BM neutrophil numbers and HSPC proliferation in BL/6 mice after alum or Gr-1 mAb injection, and in naïve Mcl-1^−^ mice (Fig. 2A–E) suggested that the mature neutrophil compartment might directly regulate the proliferation rates of hematopoietic progenitor cells. During the loss and recovery of BM neutrophils following injection of Gr-1 mAb or alum, we observed strong inverse correlations between the numbers of BM neutrophils and the frequencies of BrdU^+^ HSC (R = −0.84 to −0.94, P≤0.01, Fig. 2F), MPP (R = −0.78 to −0.94, P≤0.05), and GMP (R = −0.88 to −0.93, P≤0.01). Remarkably, an inverse relationship between BM neutrophil numbers and HSPC proliferation rates was evident not only when BM neutrophil numbers were low (days 1–2 after Gr-1 or alum, Fig. 2F) but also when BM neutrophil numbers climbed above steady-state levels (day 6, 10 µg Gr-1; days 8–12, 100 µg Gr-1; Fig. 2F). These findings are consistent with the activity of a feedback system that couples HSPC proliferation to the population density of BM neutrophils.

If BM neutrophils regulate HSPC proliferation through feedback, then changes in BM neutrophil numbers should elicit reciprocal and proportional changes in proliferation rates. We therefore injected mice with graded doses of Gr-1 mAb (0.1–100 µg) and observed dose-dependent decreases in BM neutrophil numbers on day 2, the nadir of neutrophil losses (Fig. 2G). In these mice, increased frequencies of BrdU^+^ HSC (Fig. 2G) were highly correlated (R = −0.89; P≤0.01) with the extent of neutrophil depletion. Together, these findings implicate the BM neutrophil population as an important control element in the regulation of granulopoiesis; changes in the numbers of BM neutrophils alone are sufficient to alter HSPC proliferation.

### Emergency granulopoiesis in the absence of inflammation

As noted, it is not easy to disentangle the effects of inflammation (IL-1, G-CSF, *etc*.) on granulopoiesis from those of neutrophil depletion (Fig. 2). If neutrophil depletion by Gr-1 mAb elicits significant inflammation, perhaps through complement fixation or Fc receptor signaling, then increased HSPC proliferation might not be due to reduced BM neutrophil numbers.

**Figure 3:**
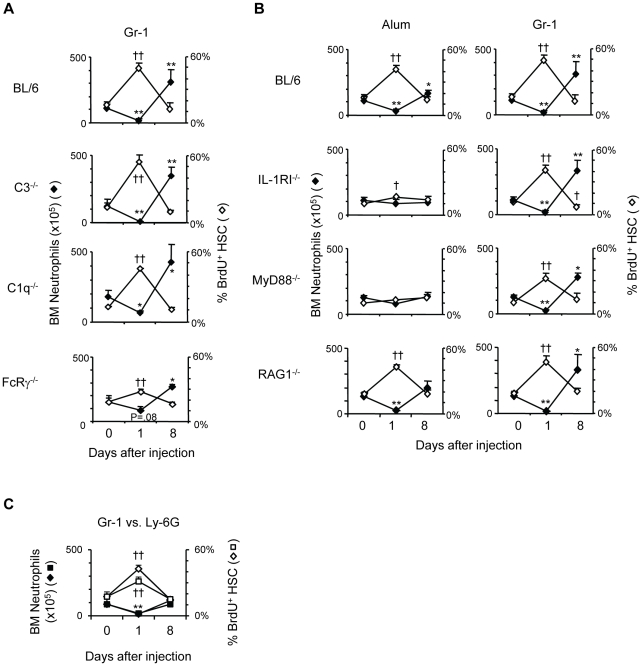
Neutrophil depletion with Gr-1 mAb elicits emergency granulopoiesis with minimal inflammation. (A) BL/6 and congenic C3^−/−^, C1q^−/−^, and FcRγ^−/−^ mice were treated with 100 µg G-1 mAb; the mean numbers(+SD) of BM neutrophils (closed) and frequencies(+SD) of BrdU^+^ HSC (open) at days 1 and 8 after treatment are shown. (B) BL/6 mice and congenic IL-1RI^−/−^, MyD88^−/−^, and RAG1^−/−^ mice were injected with alum or 100 µg Gr-1 mAb; the mean numbers(+SD) of BM neutrophils (closed) and mean frequencies(+SD) of BrdU^+^ HSC (open) at days 1 and 8 after treatment are shown. (n = 19 BL/6 mice, day 0; n = 3–8 mice for all other data points). (C) BL/6 mice were treated with 100 µg Gr-1 mAb or 100 µg Ly-6G-specific mAb (clone 1A8); the mean numbers(+SD) of BM neutrophils (closed diamonds for Gr-1 treatment, closed squares for anti-Ly-6G treatment) and frequencies(+SD) of BrdU^+^ HSC (open diamonds for Gr-1 treatment, open squares for anti-Ly-6G treatment) at days 1 and 8 after antibody injection are shown. (n = 3–5 mice for days 0 and 1, n = 2 for both treatments at day 8).

However, mice deficient for C1q [27] or C3 [28] exhibited increased HSC proliferation and neutrophil production after Gr-1 administration that were similar to congenic BL/6 mice (Fig. 3A). In FcRγ^−/−^ mice [29], the neutrophil depleting capacity of Gr-1 mAb was diminished, yet the lower reductions in BM neutrophil numbers remained associated with significant increases in HSC proliferation (day 1, P≤0.01) and rebounds in BM neutrophil numbers (day 8, P≤0.05) (Fig. 3A). Granulopoietic responses to Gr-1 treatment, therefore, are independent of complement or FcR-dependent events.

IL-1RI expression by radiation resistant cells is necessary for alum-induced emergency granulopoiesis [3]. To determine if granulopoietic responses to neutrophil depletion also depend on IL-1RI signaling, we administered Gr-1 mAb to IL-1RI^−/−^ mice; as expected [3], granulopoietic responses to alum were severely impaired in IL-1RI^−/−^ mice (Fig. 3B) but Gr-1 treatment elicited increases in HSC proliferation (day 1, P≤0.01) and BM neutrophil numbers (day 8, P≤0.01) that were identical to congenic BL/6 mice (Fig. 3B). Similarly, mice lacking MyD88, a crucial signaling component of many TLR [41] and the IL-1, IL-18 [26], and IL-33 receptors [42], did not activate emergency granulopoiesis in response to alum, but responded with increased HSC proliferation (day 1; P≤0.01) and granulopoietic output (day 8; P≤0.05) following treatment with Gr-1 mAb (Fig. 3B).

T cells have been implicated as positive regulators of granulopoiesis through the production of IL-17 [43]. However, RAG1^−/−^ mice, which lack both T and B-lymphocytes [25], had normal numbers of developing and mature neutrophils in BM (Table S1), and the frequencies of BrdU^+^ HSC, MPP, and GMP were identical to congenic BL/6 controls (Fig. 3B, unpublished data; P>0.05). Furthermore, granulopoietic responses in RAG1^−/−^ mice to both alum and Gr-1 were identical to BL/6 mice (Fig. 3B), indicating that signals from T or B lymphocytes are dispensable for HSPC responses to these treatments.

Our survey (Fig. 3A, B) of inflammatory defects provided no evidence that passive Gr-1 mAb triggers HSPC proliferation via intermediate, inflammatory pathways. Notably, HSC proliferation increased only when BM neutrophil numbers decreased, whether by mobilization in response to alum or depletion by Gr-1 mAb (Fig. 3B). We conclude that inflammation targets BM neutrophils for mobilization, but that emergency granulopoiesis is independent of IL-1RI, MyD88, or lymphocyte-derived signals.

Neutrophils express the highest amount of Gr-1 surface antigen of cells in the BM and blood (Fig. S1) but, as previously reported [44], treatment of mice with Gr-1 mAb depleted Ly-6G^-^Ly-6C^+^ monocytes (Fig. S3) in addition to neutrophils. Losses of monocytes, and not neutrophils, might account for increased HSPC proliferation after Gr-1 injection. However, mice treated with a Ly-6G-specific mAb [44] exhibited changes in hematopoiesis that were similar to those in mice treated in parallel with Gr-1 mAb: anti-Ly-6G treatment significantly reduced BM neutrophil numbers (day 1, P≤0.01) and increased frequencies of BrdU^+^ HSC (day 1, P≤0.01)(Fig. 3C). We conclude that the changes in hematopoiesis following Gr-1 administration are primarily due to the depletion of neutrophils.

### Cytokines, growth factors, and chemokines elicited by inflammation or neutropenia

Several growth factors have been implicated in emergency granulopoiesis, including G-CSF, GM-CSF, IL-3, and IL-6. We determined the concentrations of these factors and 19 other cytokines, growth factors, and chemokines (Fig. S4) in the sera of naïve Mcl-1^+^, Mcl-1^−^, and BL/6 mice, and in BL/6 animals injected with alum or Gr-1 mAb (6 h and 1-, 2-, and 8 days after treatment). The inclusion of naïve Mcl-1^−^ mice and their Mcl-1^+^ littermate controls allowed us to distinguish patterns of cytokine changes elicited by inflammation from those that are solely the consequence of neutropenia.

Of the 23 factors surveyed, the levels of 15, IL-1α/β, IL-2, IL-3, IL-4, IL-10, IL-12 p40/p70, IL-13, IL-17,GM-CSF, MIP-1α/β, RANTES, and TNFα, were identical in naïve Mcl-1^+^, Mcl-1^−^, and BL/6 mice and remained unchanged in BL/6 mice in response to alum or Gr-1 mAb (Fig. S4). The serum concentrations of three cytokines, IL-9, eotaxin, and IFNγ, fell in response to alum or Gr-1; IL-9, eotaxin, and IFNγ levels in naïve Mcl-1^+^ and Mcl-1^−^ mice were also low and not significantly different (Fig. S4).

**Figure 4:**
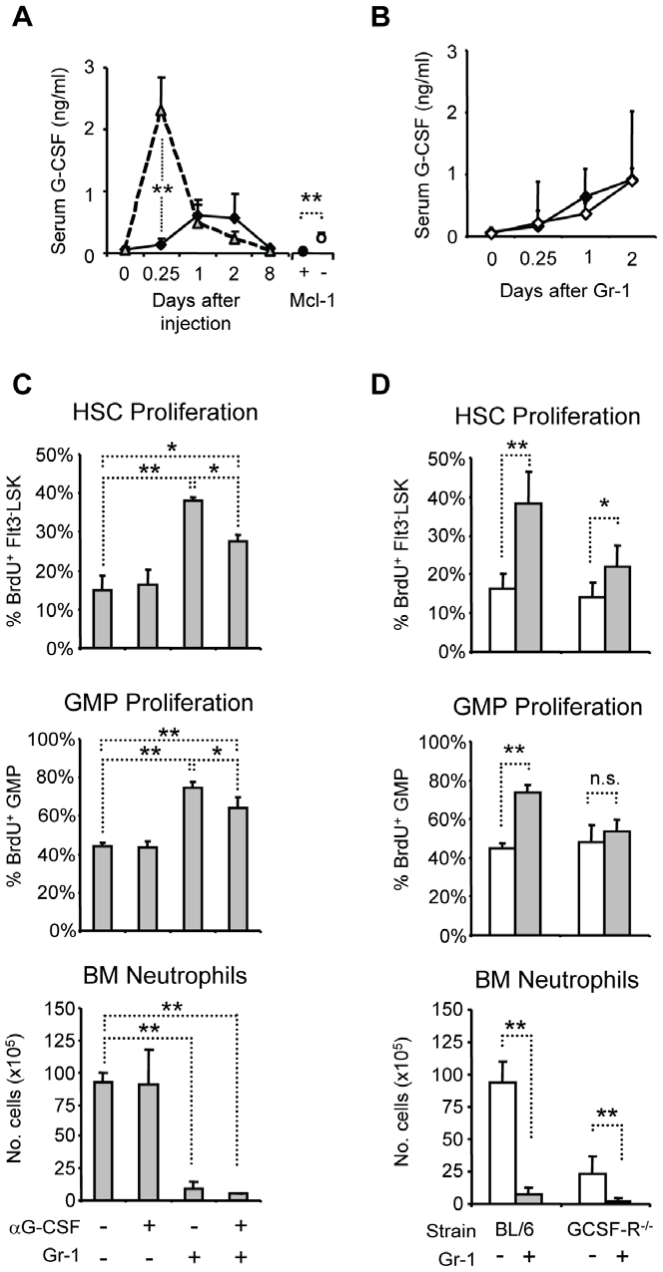
Neutrophil depletion elicits G-CSF-dependent and -independent HSPC proliferation. (A) Serum from naïve BL/6, Mcl-1^+^ (closed circle), Mcl-1^−^ (open circle) mice, and BL/6 mice treated with alum (open triangles) or Gr-1 mAb (closed diamonds) was analyzed for G-CSF in a multiplex bead array. The mean concentrations(+SD) of G-CSF/ml of serum are shown (n = 3–7). (B) Serum G-CSF in BL/6 (closed) and IL-1RI^−/−^ (open) mice after Gr-1 administration; the mean concentrations(+SD) of G-CSF/ml are shown (n = 3–5). (C) Effect of G-CSF neutralization on proliferative response of HSPC to induced neutropenia. BL/6 mice were injected or not with anti-GCSF, then administered Gr-1 mAb. The mean frequencies(+SD) of BrdU^+^ cells in the HSC and GMP compartments, and the numbers(+SD) of BM neutrophils on day 1 after treatment are shown (n = 4–5). (D) BL/6 and G-CSF-R^−/−^ mice were treated or not with Gr-1 mAb, then sacrificed 24 hours later for analysis. The mean frequencies(+SD) of BrdU^+^ cells in the HSC and GMP compartments, and the mean numbers(+SD) of BM neutrophils are shown (n = 3–9). *, P≤0.05; **, P≤0.01.

Alum increased serum IL-5, IL-6, KC, MCP-1 (Fig. S4), and G-CSF (Fig. 4A); the serum levels of these cytokines peaked at 6 h after injection and then returned to normal (Fig. S4). Gr-1 administration elicited only KC, MCP-1 (Fig. S4), and G-CSF (Fig. 4A). In contrast to the rapid rise in serum G-CSF elicited by alum, there was no increase in G-CSF 6 h after Gr-1 injection (Fig. 4A) but G-CSF levels gradually increased to those observed in alum-treated mice on days 1 and 2 before returning to control levels on day 8 (Fig. 4A). In neutropenic Mcl-1^−^ mice, G-CSF (Fig. 4A) and KC (Fig. S4) were also constitutively elevated compared to control littermates.

The modest and delayed increases in G-CSF observed after Gr-1 injection and the constitutive G-CSF in naive Mcl-1^−^ mice might represent a direct consequence of neutropenia (Fig. 4A).Whereas alum did not elicit G-CSF in the absence of IL-1RI signals (Fig. 1A), Gr-1 depletion elicited G-CSF equally in BL/6 and IL-1RI^−/−^ mice (Fig. 4B), indicating that G-CSF can be induced through two distinct pathways: an IL-1RI-dependent inflammatory pathway and an IL-1RI-independent pathway activated by neutropenia. We conclude that IL-1RI signals rapidly induce G-CSF and BM neutrophil mobilization, but that G-CSF levels are sustained as a result of BM neutropenia.

### Neutropenia elicits G-CSF dependent and independent increases in HSPC proliferation

G-CSF's capacity to modulate BM neutrophil numbers by controlling their migration raised the possibility that G-CSF's effects on HSPC proliferation might be indirect. To determine if G-CSF/G-CSF-R signals are required for proliferative HSPC responses to neutropenia, we injected mice with G-CSF neutralizing mAb then administered Gr-1 mAb. With G-CSF neutralization, the effects of Gr-1 depletion on HSC and GMP proliferation were reduced, but the frequencies of BrdU^+^ HSC and GMP were still increased over naïve controls (P≤0.05, Fig. 4C), indicating that a portion of the HSPC response to BM neutropenia is G-CSF-dependent.

We next examined the effects of Gr-1 depletion on G-CSF-R^−/−^ mice. Gr-1 mAb depleted the residual BM neutrophils in G-CSF-R^−/−^ mice but failed to elicit an increase in GMP proliferation (Fig. 4D). However, the frequency of BrdU^+^ HSC rose modestly but significantly after Gr-1 injection, indicating that neutropenia activates a G-CSF-R-independent mechanism for increased HSC proliferation (Fig. 4D).

### C/EBPβ is dispensable for increased HSPC proliferation during emergency granulopoiesis

C/EBPβ is thought to be necessary for a distinct pathway of granulopoiesis elicited by inflammatory signals [2]. However, we observed that neutropenia increased HSPC proliferation and neutrophil production similar to alum, but without evidence of inflammation (Fig. 2 and 3). To determine if C/EBPβ is necessary for granulopoietic responses to neutropenia, we depleted neutrophils in C/EBPβ^−/−^ mice and C/EBPβ-sufficient littermates with Gr-1 mAb. Hematopoiesis in C/EBPβ^+/+^ and C/EBPβ^+/−^ mice was identical; data from these mice were pooled and are referred to hereafter as C/EBPβ^+^ mice.

**Figure 5:**
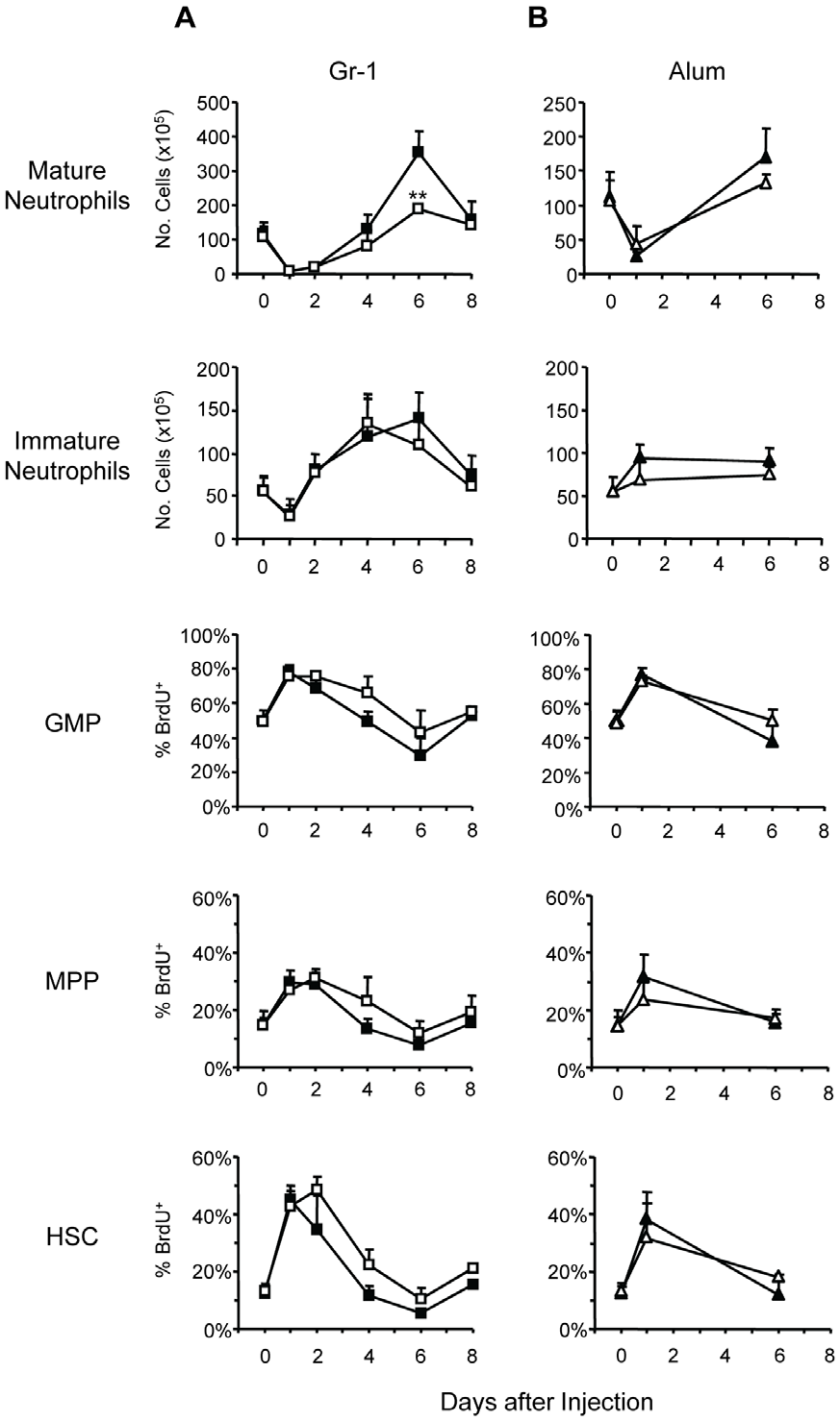
C/EBPβ is dispensable for proliferative responses of HSPC to Gr-1 administration or alum immunization. C/EBPβ^−/−^ mice (open) and control littermates (closed) were injected with 10 µg Gr-1 mAb (A) or alum (B). The mean numbers(+SD) of mature and immature neutrophils (Fig. S1) in BM, and mean frequencies(+SD) of BrdU^+^ cells in the HSC, MPP, and GMP compartments at different intervals after treatment are shown. Significant differences between C/EBPβ^+^ and C/EBPβ^−/−^ mice at corresponding intervals are indicated (day 0, n = 12 for C/EBPβ^+^ mice and n = 7 for C/EBPβ^−/−^ mice; for all others, n = 3–5 mice). **, P≤0.01.

C/EBPβ^−/−^ mice had equivalent numbers of BM neutrophils as C/EBPβ^+^ mice (Fig. 5A), consistent with previous reports [45], and neutrophils were similarly depleted by Gr-1 mAb (Fig. 5A). However, the supranormal recovery of BM neutrophils was blunted in mice lacking C/EBPβ (Fig. 5A); BM neutrophil numbers peaked on day 6 only modestly higher than in untreated mice (1.7-fold, P≤0.05), and were only half of that in C/EBPβ^+^ mice at the same interval (P≤0.01) (Fig. 5A). Thus, C/EBPβ is required for optimal granulopoietic responses to neutropenia.

C/EBPβ is thought to allow for increased proliferation of GMP in response to growth factors [2] but, surprisingly, C/EBPβ^−/−^ mice exhibited similar increases in frequencies of BrdU^+^ HSC, MPP, and GMP as C/EBPβ^+^ mice (Fig. 5A). Although ANOVA indicated significant differences in the proliferative responses of HSC and GMP, but not MPP, in C/EBPβ^+^ and C/EBPβ^−/−^ mice (P≤0.05), a Tukey post-hoc test failed to reveal statistical significance in pair-wise comparisons at individual time points. Notably, the frequencies of BrdU^+^ HSC and GMP were slightly higher in C/EBPβ^−/−^ mice than in C/EBPβ^+^ mice at day 4 after Gr-1 treatment (Fig. 5A), although we are uncertain of the biological relevance of this modest, non-significant increase. We found no evidence that C/EBPβ deficiency restricts the proliferative capacity of HSC, MPP, or GMP.

The immature neutrophil population, comprising myelocytes and metamyelocytes (SSC^int^Ly-6B^int^Gr-1^int^CD11b^+^, Fig. S1), underwent identical changes in C/EBPβ^+^ and C/EBPβ^−/−^ mice after Gr-1 treatment (Fig. 5A), indicating equivalent expansions of progenitor compartments and cellular delivery into the neutrophil lineage. These observations dissociate C/EBPβ from the proliferative effects of growth factors on HSPC; instead, the lack of rebounding neutrophilias in C/EBPβ^−/−^ mice stems from a defect at the transition between immature and mature neutrophils.

Interestingly, C/EBPβ was dispensable for granulopoietic responses to alum. Alum elicited similar increases in HSPC proliferation in C/EBPβ^+^ and C/EBPβ^−/−^ mice (Fig. 5B). The number of mature neutrophils in BM of C/EBPβ^−/−^ mice on day 6, although modestly lower, was not significantly different from control mice (Fig. 5B). Since Gr-1 administration elicits a more robust granulopoietic response than alum (Fig. 2B), C/EBPβ-independent pathways of neutrophil differentiation must suffice for the replenishment of mature neutrophils after immunization.

## Discussion

Microbial infection and/or tissue damage elicit growth factors that can accelerate granulopoiesis to expedite the elimination of pathogens and necrotic cells [46]. Recent studies have suggested that “emergency” or “demand-driven” granulopoiesis represents a developmental pathway distinct from the steady-state hematopoiesis that normally sustains neutrophil pools [2]. Our findings, however, indicate that the depletion of neutrophil reserves during acute inflammation activates a feedback mechanism that increases G-CSF production/availability and accelerates neutrophil production. We propose that feedback mechanisms that maintain neutrophil pools in the steady-state also play critical roles in the accelerated granulopoiesis associated with inflammation.

We have shown that alum induces emergency granulopoiesis via IL-1RI-dependent signals [3]. Notably, defective emergency granulopoiesis in IL-1RI^−/−^ mice was accompanied by failed mobilization of BM neutrophils [3]. Now we demonstrate that alum triggers IL-1RI-dependent activation of G-CSF (Fig. 1A), and that G-CSF/G-CSF-R signals are necessary for both the proliferative responses of HSPC and the mobilization of BM neutrophils (Fig. 1B-E).

That HSPC proliferation and neutrophil mobilization are tightly linked in IL-1RI- and G-CSF-R deficient mice suggested that changes in HSPC proliferation and granulopoiesis in response to inflammation might reflect the common reduction in BM neutrophil reserves. Evidence consistent with this sort of feedback control have been offered previously [6], [7], [8], and most mathematical models for granulopoietic regulation contain feedback components linking neutrophil population density to proliferation and differentiation by neutrophil precursors [10], [11], [12], [13]. Recent studies have suggested that IL-17-producing T cells modulate G-CSF production as part of a feedback mechanism that regulates granulopoiesis [47]. In our hands, however, the BM of RAG1^−/−^ and control mice contained equivalent numbers of neutrophils (Table S1) and RAG1^−/−^ mice exhibited normal granulopoietic responses to alum or passive Gr-1 antibody (Fig. 3B). Studies of RAG1^−/−^ mice do not rule out lymphoid-tissue inducer cells or double negative 1 T cells as sources of IL-17 for granulopoietic regulation [48], [49], but mature T cells are unnecessary for steady-state and inflammatory control of BM neutrophil numbers. We note also that IL-17 serum levels in BL/6 mice given alum or Gr-1 antibody were not different from naïve controls, Mcl-1^−^, or Mcl-1^+^ mice (Fig. S4).

To determine the contribution of feedback to emergency granulopoiesis, we compared the effects of neutropenia to those elicited by inflammation by tracking simultaneously HSPC proliferation and neutrophil production. BM neutropenia was invariably coupled to increased HSPC proliferation, regardless of how neutrophil numbers were reduced (Fig. 2). Neutrophil depletion with Gr-1 mAb induced rapid increases in HSC, MPP, and GMP proliferation followed by supranormal BM neutrophil numbers (Fig. 2); mice rendered chronically neutropenic by inactivation of Mcl-1 in myeloid cells [30] had constitutively elevated rates of HSPC proliferation (Fig. 2C-E) and a surplus of immature BM neutrophils (Table S1).

Significantly, increased HSPC proliferation in Mcl-1^−^ mice was only associated with the absence of mature neutrophils; all other hematopoietic lineages in Mcl-1^−^ mice equaled (or were greater than) that of littermate controls (Table S1). Given that HSPC proliferation in lymphopenic RAG1^−/−^ mice was indistinguishable from controls (Fig. 3B), we conclude that the link between mature BM neutrophils and HSPC proliferation is specific. Mature neutrophils in BM actively control the pace of granulopoiesis by regulating HSPC proliferation.

Following alum or Gr-1 administration, and in Mcl-1^−^ mice, BM neutropenia was associated with lesser or greater increases in serum G-CSF (Fig. 4A), a cytokine with pleiotropic effects [15], [16]. Whereas alum induced sharp, IL-1RI-dependent increases in G-CSF followed by longer periods of modest G-CSF elevation (Fig. 4A), the neutropenia induced by Gr-1 mAb or Mcl-1 deficiency was associated only with the modest G-CSF plateau (Fig. 4A). These distinct kinetics suggest multiple pathways of G-CSF induction: one driven by inflammation and the second by neutropenia itself. Whereas alum failed to induce G-CSF in IL-1RI^−/−^ mice (Fig. 1A), neutropenia induced by Gr-1 mAb elicited G-CSF equally in BL/6 and IL-1RI^−/−^ mice (Fig. 4B). We conclude that alum elicits a rapid increase in G-CSF via IL-1RI to mobilize BM neutrophil reserves; the sustained plateau of elevated G-CSF, however, is a consequence of BM neutropenia.

Mice lacking G-CSF-R are neutropenic (Fig. 1D, 4C and [16]) but, in contrast to neutropenic Mcl-1^−^ mice, HSPC proliferation in G-CSF-R^−/−^ mice was normal, indicating that HSC and GMP are insensitive to neutrophil population density in the absence of G-CSF-R. However, depletion of the residual BM neutrophils in G-CSF-R^−/−^ mice induced a modest, but significant increase in HSC (but not GMP) proliferation (Fig. 4D). Thus the G-CSF/G-CSF-R axis is an important mediator of density-dependent feedback between BM neutrophils and HSPC, but an additional, G-CSF-R independent feedback signal acts specifically on HSC.

We suggest that BM neutrophils suppress HSPC proliferation by regulating the availability of G-CSF (and perhaps other growth factors) to drive HSPC proliferation and differentiation; the strength of suppression is proportional to the numbers of mature neutrophils in BM. By mobilizing neutrophils, inflammation activates this feedback mechanism to increase HSPC proliferation and neutrophil output. As granulopoiesis accelerates, the number of mature neutrophils in BM increases, reestablishing a suppressive environment and eventually, the steady-state equilibrium.

Recent studies concluded that the transcription factors C/EBPα and C/EBPβ operate in GMP as developmental switches for independent granulopoietic pathways, and that the absence of reactive neutrophilia in C/EBPβ^−/−^ mice is the consequence of defective HSPC proliferation [2]. Our observations, however, indicate that proliferative responses of C/EBPβ^−/−^ HSPC to neutrophil depletion or alum-induced inflammation are normal (Fig. 5). Instead, in C/EBPβ^−/−^ mice, the final maturation of immature neutrophils is impaired during granulopoietic recovery from Gr-1 treatment (Fig. 5A). Our data suggest that C/EBPα and C/EBPβ operate at distinct stages of granulopoiesis, with C/EBPα directing the differentiation of CMP to GMP [4], and C/EBPβ acting at terminal neutrophil differentiation. C/EBPβ-dependent processes are necessary for the rapid and efficient generation of mature neutrophils during emergency granulopoiesis, but are not rate-limiting in steady-state conditions, as BM neutrophil reserves are intact in naïve C/EBPβ^−/−^ mice (Fig. 5).

We conclude that regulatory feedback is an important source of signals for the induction of emergency granulopoiesis, a view that can explain patterns of HSPC proliferation that do not fit standard granulopoietic models. For example, the lack of HSPC proliferation in response to vaccinia virus in MyD88^−/−^ mice [50] might be a consequence of impaired neutrophil mobilization. Conversely, pathogens or agents that drain the BM neutrophil reserve will trigger G-CSF production and increase HSPC proliferation. Evaluations of growth factors and/or microbial products on HSPC proliferation and granulopoiesis must consider the effects of neutrophil mobilization.

Over the last 50 years, various mathematical models have been developed to describe the changes in granulopoiesis following the perturbation of neutrophil compartments. Our observations are remarkably consistent with models that posit the BM neutrophil compartment as the primary variable for the regulation of granulopoiesis [10], [11]. Our findings, however, imply that the regulatory effects of BM neutrophil population density extend to the accelerated granulopoiesis associated with inflammation.

## Supporting Information

Figure S1 **Flow cytometric definitions of bone marrow leukocytes.** Ter119^−^ cells in BM were divided into four populations (R1–R4) based on side-scatter properties and staining with Ly-6B mAb. Flow cytometric definitions of lymphocytes (R1), eosinophils (R2), neutrophils (R3), and monocytes (R4) were based on the expression Gr-1, Ly-6G, Ly-6C, CD11b, CCR3, CX3CR1-GFP, TCRβ, and B220 and were confirmed by histological examination of sorted cells. Neutrophil lineage cells (R3) were subdivided into three populations based on expression of CD11b and Ly-6G or Gr-1. Cells in R5 expressed the progenitor markers CD31, CD34 and c-Kit, and exhibited morphological features of myeloblasts and promyelocytes. Cells in R6 lost CD31 and CD34 and had low c-Kit staining; histologically, they were classified as myelocytes and metamyelocytes. Cells in R7 did not express the progenitor markers CD31, CD34, or c-Kit and exhibited histological features of band and segmented neutrophils.
(TIF)

Figure S2 **Effects of adjuvant immunization and Gr-1 administration on HSPC numbers.** BL/6 mice were injected *i.p.* with 10 µg Gr-1 (open squares), 100 µg Gr-1 (closed diamonds), or an alum/antigen mixture (shaded triangles). BM cells of the hindlimbs were analyzed at different intervals by flow cytometry, and the numbers of HSC, MPP, and GMP were determined. The mean(+SD) numbers of cells in the femurs and tibiae at each interval are shown (day 0, n = 19; for others, n = 3–10). In the right panel, the numbers of HSC, MPP, and GMP in the BM of Mcl-1-sufficient and deficient mice are shown.
(TIF)

Figure S3 **Effects of adjuvant immunization and Gr-1 administration on monocytes, eosinophils, B-lineage cells, and erythroid lineage cells in BM.** BL/6 mice were injected *i.p.* with 10 µg Gr-1 (open squares), 100 µg Gr-1 (closed diamonds), or an alum/antigen mixture (shaded triangles). BM cells of the hindlimbs were analyzed at different intervals by flow cytometry, and the numbers of monocytes, eosinophils, B-lineage cells (B220^+^), and erythroid lineage cells (Ter119^+^) were determined. The mean(+SD) numbers of cells in the femurs and tibiae at each interval are shown (day 0, n = 19; for others, n = 3–10). Significant differences from naïve mice are shown for treatment with 10 µg Gr-1 (*, P≤0.05 and **, P≤0.01), treatment with 100 µg Gr-1 (†, P≤0.05 and ††, P≤0.01), and immunization with alum (^#^, P≤0.05 and ^##^, P≤0.01).
(TIF)

Figure S4 **Serum cytokines in inflamed and neutropenic mice.** (A) The concentrations of cytokines in sera of BL/6 mice immunized with alum (shaded triangles) or treated with 100 µg Gr-1 mAb (closed diamonds) on days 0, 0.25, 1, 2, and 8 were determined using a multiplex bead array (n = 4–5 mice per data point). (B) Serum concentrations of cytokines in control Mcl-1^+^ mice (open, n = 3) and neutropenic Mcl-1^−^ mice (shaded, n = 7) are shown. The dotted horizontal line in each graph represents the lowest concentration of confident detection.
(TIF)

Table S1 **Numbers of BM leukocytes (x10^5^) in neutrophil deficient mice (Mcl-1** ^−^ **) and control littermates (Mcl-1^+^), and in RAG1** ^−**/**−^ **mice and congenic C57BL/6 mice.** The mean numbers(±SD) (x10^5^) of each leukocyte type in the femurs and tibiae of Mcl-1^−^ and congenic Mcl-1^+^ mice, and in RAG1^−/−^ and congenic BL/6 mice are shown. See Figure S1 for flow cyometric definitions of B-lineage cells, eosinophils, monocytes, and neutrophil subpopulations. Statistical significance between the numbers of cells in knockout mice and congenic controls was determined by Student's *t*-test; (n = 5 for Mcl-1^+^ mice, n = 10 for Mcl-1^−^ mice; n = 19 for BL/6 mice; n = 4 for RAG1^−/−^ mice).
(DOC)
