# Supplementary figures and images for: Inflammation Triggers Emergency Granulopoiesis through a Density-Dependent Feedback Mechanism
Source: PLoS One. 2011 May 31;6(5):e19957. doi: 10.1371/journal.pone.0019957 (PMC3104996; doi:10.1371/journal.pone.0019957)

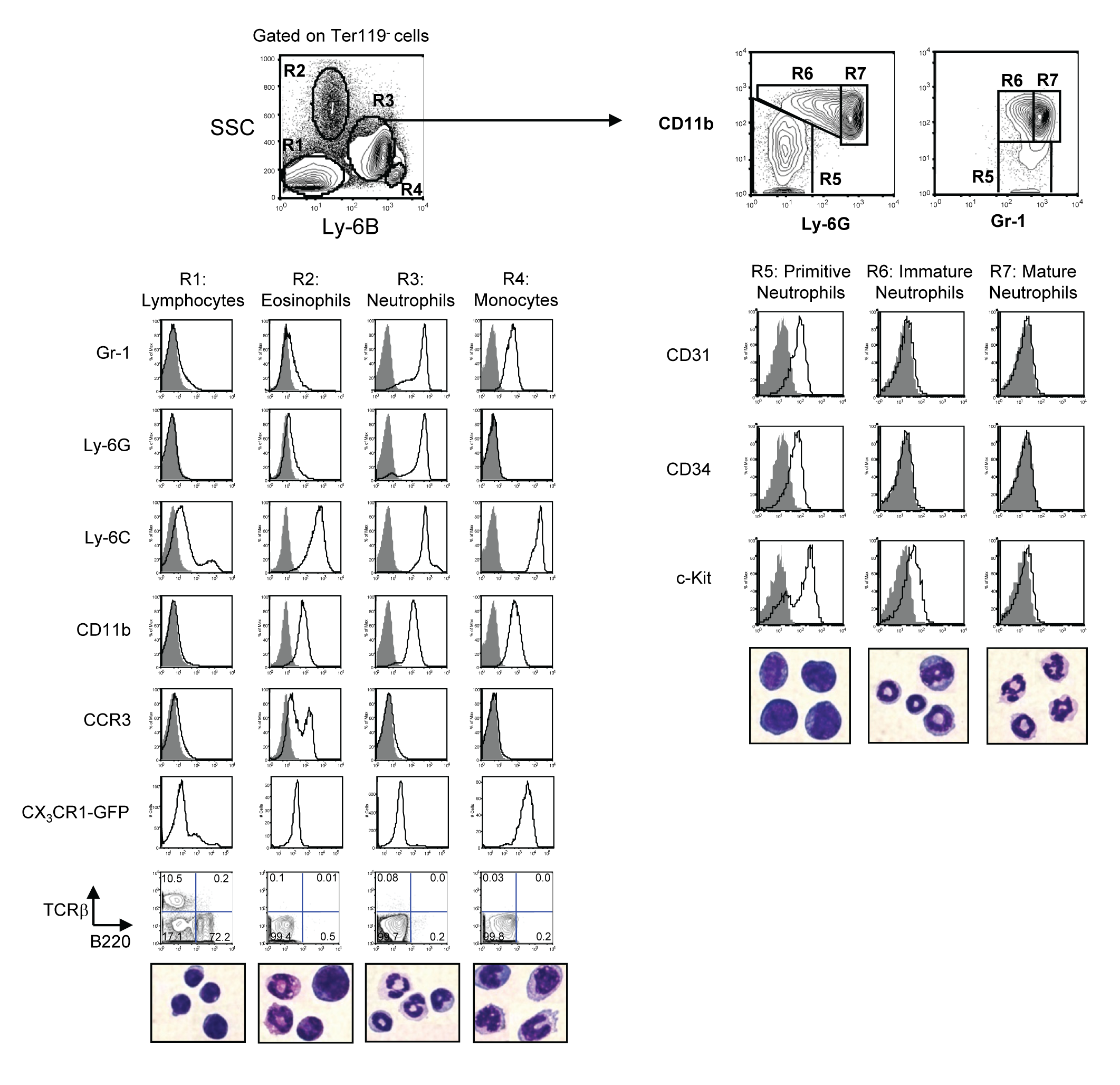

Supplement: Figure S1 — Flow cytometric definitions of bone marrow leukocytes. Ter119− cells in BM were divided into four populations (R1–R4) based on side-scatter properties and staining with Ly-6B mAb. Flow cytometric definitions of lymphocytes (R1), eosinophils (R2), neutrophils (R3), and monocytes (R4) were based on the expression Gr-1, Ly-6G, Ly-6C, CD11b, CCR3, CX3CR1-GFP, TCRβ, and B220 and were confirmed by histological examination of sorted cells. Neutrophil lineage cells (R3) were subdivided into three populations based on expression of CD11b and Ly-6G or Gr-1. Cells in R5 expressed the progenitor markers CD31, CD34 and c-Kit, and exhibited morphological features of myeloblasts and promyelocytes. Cells in R6 lost CD31 and CD34 and had low c-Kit staining; histologically, they were classified as myelocytes and metamyelocytes. Cells in R7 did not express the progenitor markers CD31, CD34, or c-Kit and exhibited histological features of band and segmented neutrophils. (TIF) [file pone.0019957.s001.tif]

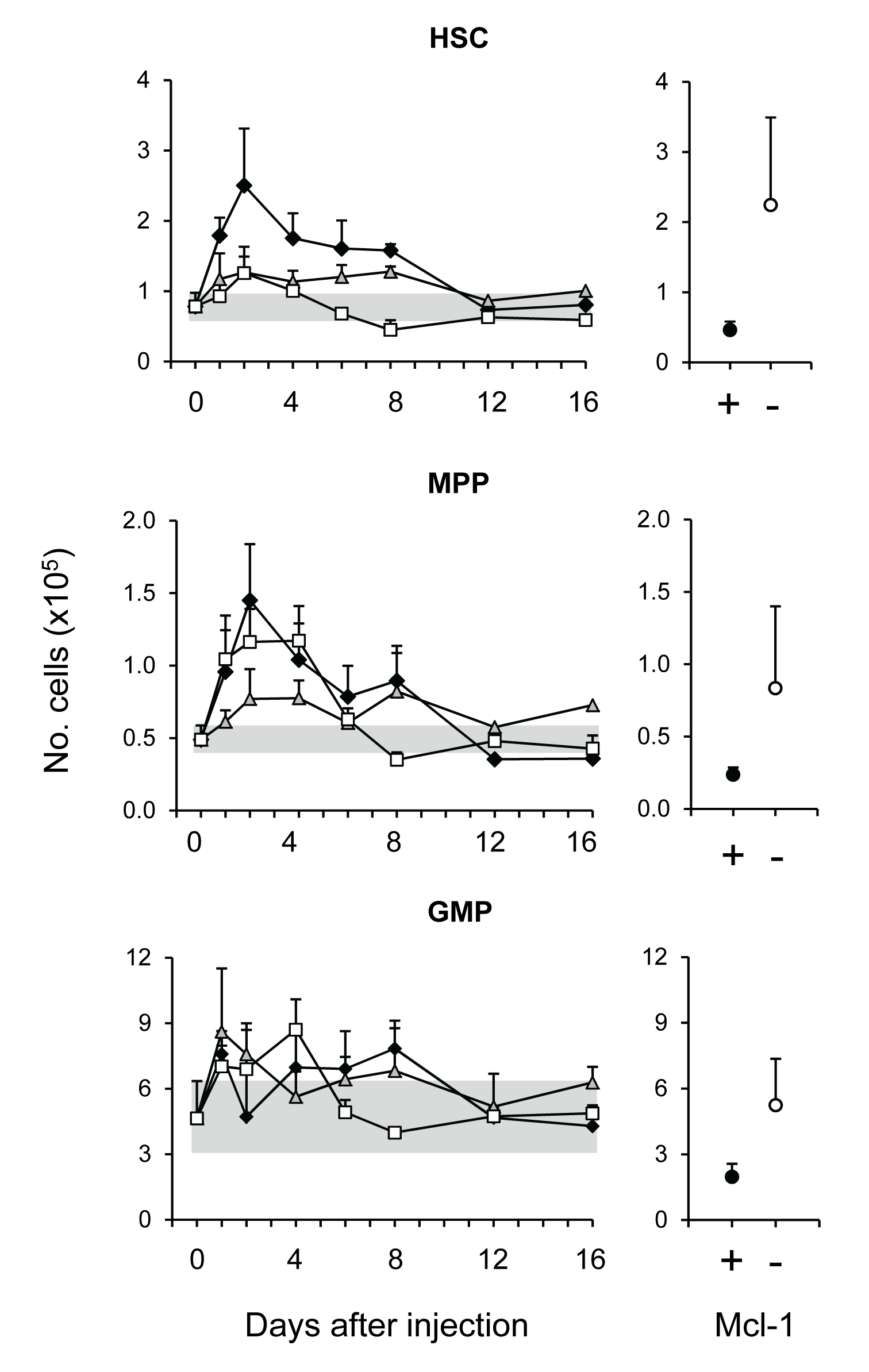

Supplement: Figure S2 — Effects of adjuvant immunization and Gr-1 administration on HSPC numbers. BL/6 mice were injected i.p. with 10 µg Gr-1 (open squares), 100 µg Gr-1 (closed diamonds), or an alum/antigen mixture (shaded triangles). BM cells of the hindlimbs were analyzed at different intervals by flow cytometry, and the numbers of HSC, MPP, and GMP were determined. The mean(+SD) numbers of cells in the femurs and tibiae at each interval are shown (day 0, n = 19; for others, n = 3–10). In the right panel, the numbers of HSC, MPP, and GMP in the BM of Mcl-1-sufficient and deficient mice are shown. (TIF) [file pone.0019957.s002.tif]

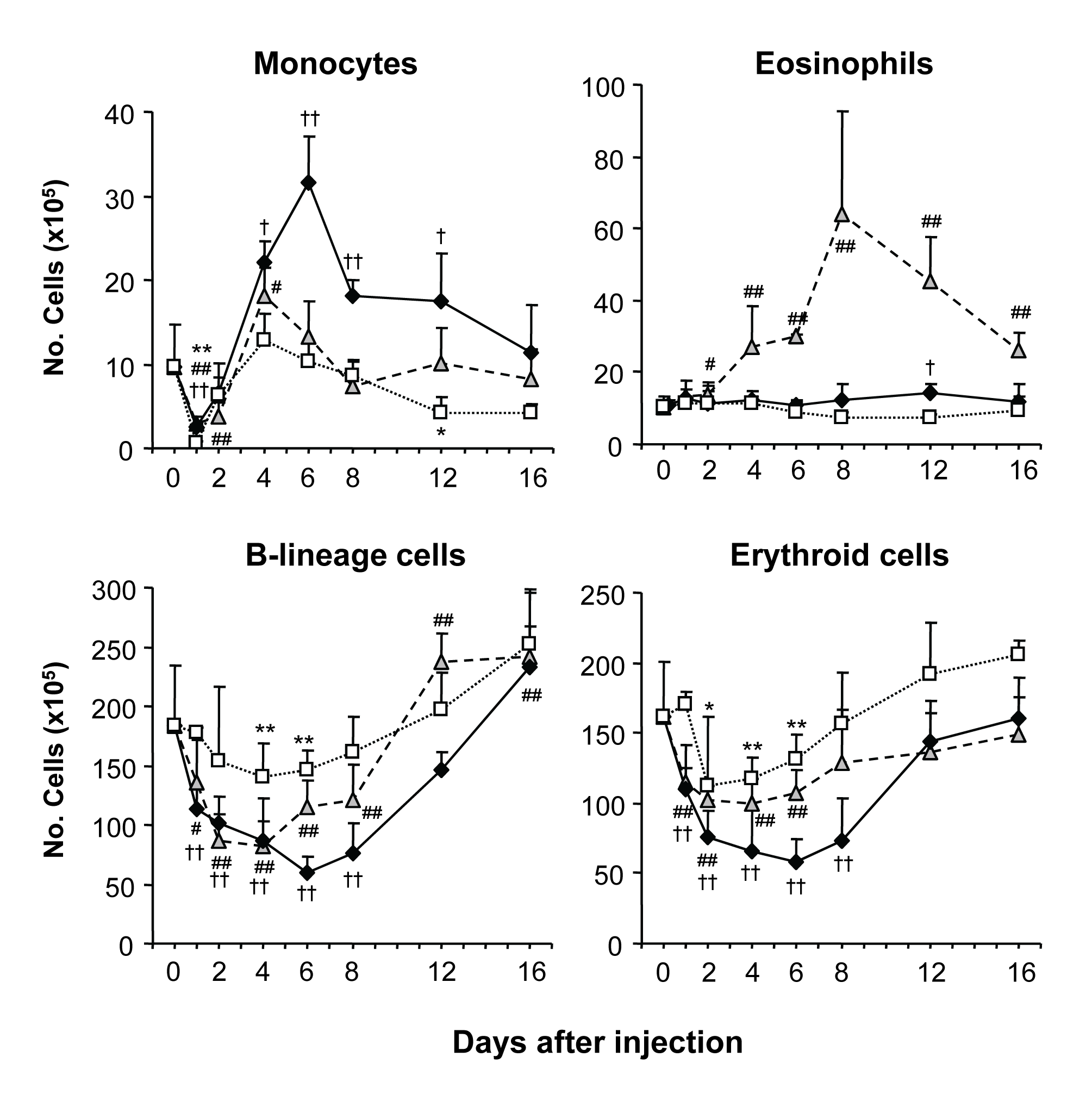

Supplement: Figure S3 — Effects of adjuvant immunization and Gr-1 administration on monocytes, eosinophils, B-lineage cells, and erythroid lineage cells in BM. BL/6 mice were injected i.p. with 10 µg Gr-1 (open squares), 100 µg Gr-1 (closed diamonds), or an alum/antigen mixture (shaded triangles). BM cells of the hindlimbs were analyzed at different intervals by flow cytometry, and the numbers of monocytes, eosinophils, B-lineage cells (B220+), and erythroid lineage cells (Ter119+) were determined. The mean(+SD) numbers of cells in the femurs and tibiae at each interval are shown (day 0, n = 19; for others, n = 3–10). Significant differences from naïve mice are shown for treatment with 10 µg Gr-1 (*, P≤0.05 and **, P≤0.01), treatment with 100 µg Gr-1 (†, P≤0.05 and ††, P≤0.01), and immunization with alum (#, P≤0.05 and ##, P≤0.01). (TIF) [file pone.0019957.s003.tif]

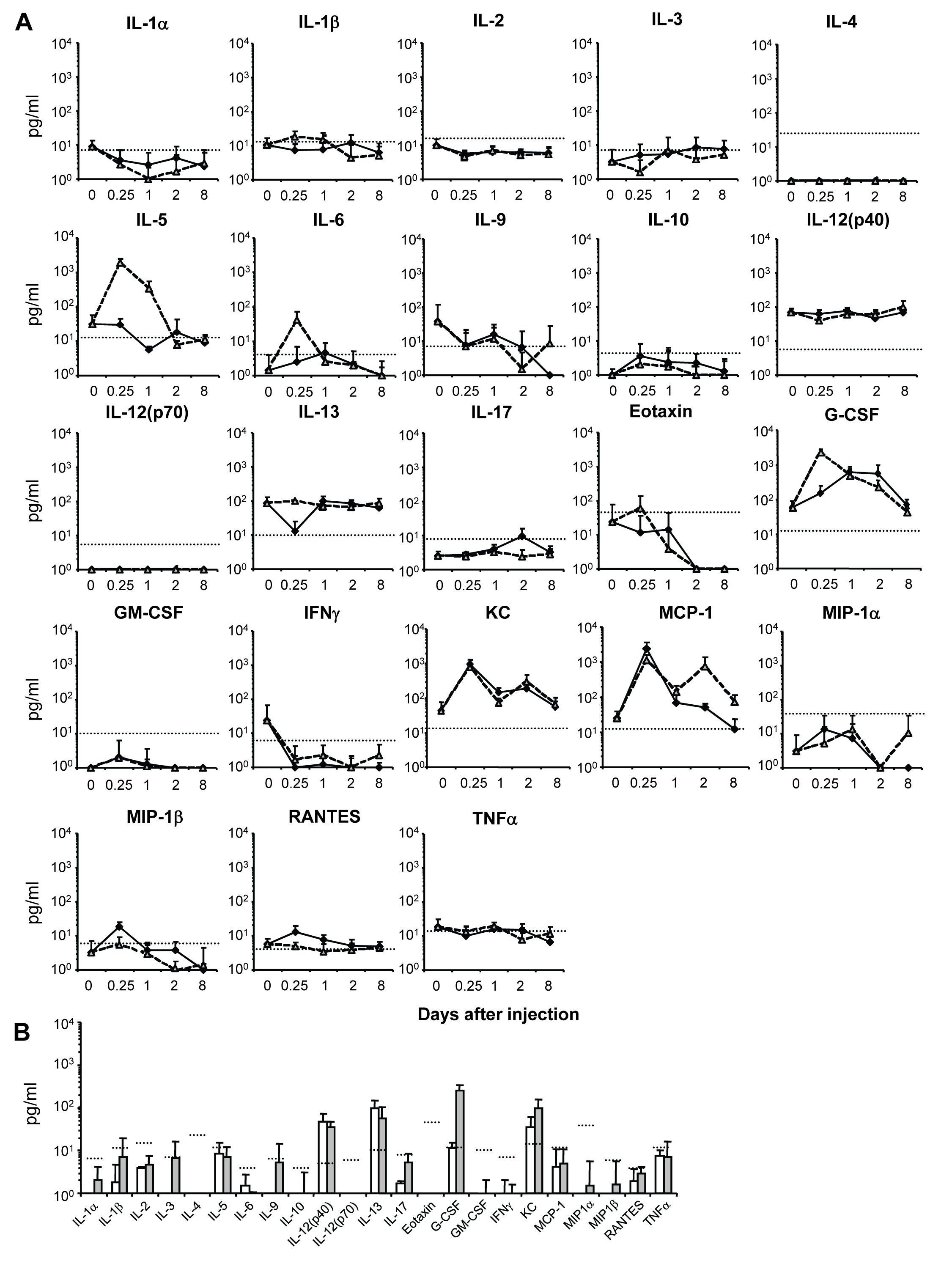

Supplement: Figure S4 — Serum cytokines in inflamed and neutropenic mice. (A) The concentrations of cytokines in sera of BL/6 mice immunized with alum (shaded triangles) or treated with 100 µg Gr-1 mAb (closed diamonds) on days 0, 0.25, 1, 2, and 8 were determined using a multiplex bead array (n = 4–5 mice per data point). (B) Serum concentrations of cytokines in control Mcl-1+ mice (open, n = 3) and neutropenic Mcl-1− mice (shaded, n = 7) are shown. The dotted horizontal line in each graph represents the lowest concentration of confident detection. (TIF) [file pone.0019957.s004.tif]
