# Supplementary material for: Inflammation Triggers Emergency Granulopoiesis through a Density-Dependent Feedback Mechanism
Source: PLoS One. 2011 May 31;6(5):e19957. doi: 10.1371/journal.pone.0019957 (PMC3104996; doi:10.1371/journal.pone.0019957)
Supplement: Table S1 — Numbers of BM leukocytes (x105) in neutrophil deficient mice (Mcl-1 − ) and control littermates (Mcl-1+), and in RAG1 −/− mice and congenic C57BL/6 mice. The mean numbers(±SD) (x105) of each leukocyte type in the femurs and tibiae of Mcl-1− and congenic Mcl-1+ mice, and in RAG1−/− and congenic BL/6 mice are shown. See Figure S1 for flow cyometric definitions of B-lineage cells, eosinophils, monocytes, and neutrophil subpopulations. Statistical significance between the numbers of cells in knockout mice and congenic controls was determined by Student's t-test; (n = 5 for Mcl-1+ mice, n = 10 for Mcl-1− mice; n = 19 for BL/6 mice; n = 4 for RAG1−/− mice). (DOC) [file pone.0019957.s005.doc]

| **Cell Type** | **Mcl-1^+^** | **Mcl-1^-^** | **t-Test (P)** | **C57BL/6** | **RAG1^-/-^** | **t-Test (P)** |
| --- | --- | --- | --- | --- | --- | --- |
| Total cells | 330.8±70.0 | 313.7±66.7 | 0.663 | 614.3 ± 102.2 | 560.0 ± 114.5 | 0.429 |
| Erythroid (Ter119^+^) | 64.8 ± 16.1 | 70.7 ± 26.5 | 0.598 | 162.2 ± 38.9 | 198.9 ± 40.5 | 0.168 |
| B-lineage (B220^+^) | 68.6 ± 14.0 | 56.0 ± 21.2 | 0.198 | 184.0 ± 49.7 | 76.6 ± 33.2 | 0.002 |
| Eosinophils | 7.5 ± 4.1 | 10.5 ± 5.8 | 0.267 | 10.4 ± 3.1 | 9.7 ± 3.7 | 0.745 |
| Monocytes | 16.5 ± 1.8 | 33.8 ± 17.0 | 0.010 | 9.6 ± 5.2 | 11.7 ± 3.6 | 0.371 |
| Primitive Neutrophils | 5.9 ± 1.1 | 12.9 ± 6.0 | 0.025 | 10.9 ± 4.0 | 13.9 ± 4.8 | 0.307 |
| Immature Neutrophils | 29.5 ± 6.1 | 66.8 ± 32.1 | 0.025 | 54.0 ± 21.8 | 66.8 ± 14.3 | 0.191 |
| Mature Neutrophils | 104.8 ± 28.6 | 18.7 ± 9.3 | 0.002 | 110.6 ± 31.6 | 133.7 ± 30.2 | 0.232 |

**Table S1.** Numbers of BM leukocytes (x10^5^) in neutrophil deficient mice (Mcl-1^-^) and control littermates (Mcl-1^+^), and in RAG1^-/-^ mice and congenic C57BL/6 mice.

The mean numbers(±SD) (x10^5^) of each leukocyte type in the femurs and tibiae of Mcl-1^-^ and congenic Mcl-1^+^ mice, and in RAG1^-/-^ and congenic BL/6 mice are shown. See Figure S1 for flow cyometric definitions of B-lineage cells, eosinophils, monocytes, and neutrophil subpopulations. Statistical significance between the numbers of cells in knockout mice and congenic controls was determined by Student’s *t*-test; (n=5 for Mcl-1^+^ mice, n=10 for Mcl-1^-^ mice; n=19 for BL/6 mice; n=4 for RAG1^-/-^ mice).
